# Synergistic Effects of Toxic Elements on Heat Shock Proteins

**DOI:** 10.1155/2014/564136

**Published:** 2014-07-20

**Authors:** Khalid Mahmood, Saima Jadoon, Qaisar Mahmood, Muhammad Irshad, Jamshaid Hussain

**Affiliations:** ^1^Department of Biology, Government Post-Graduate College Asghar Mall, Rawalpindi, Pakistan; ^2^Department of Natural Resource Engineering and Management, University of Kurdistan-Hawler Erbil, Kurdistan Region, Iraq; ^3^Department of Environmental Sciences, COMSATS Institute of Information Technology, Abbottabad 22060, Pakistan

## Abstract

Heat shock proteins show remarkable variations in their expression levels under a variety of toxic conditions. A research span expanded over five decades has revealed their molecular characterization, gene regulation, expression patterns, vast similarity in diverse groups, and broad range of functional capabilities. Their functions include protection and tolerance against cytotoxic conditions through their molecular chaperoning activity, maintaining cytoskeleton stability, and assisting in cell signaling. However, their role as biomarkers for monitoring the environmental risk assessment is controversial due to a number of conflicting, validating, and nonvalidating reports. The current knowledge regarding the interpretation of HSPs expression levels has been discussed in the present review. The candidature of heat shock proteins as biomarkers of toxicity is thus far unreliable due to synergistic effects of toxicants and other environmental factors. The adoption of heat shock proteins as “suit of biomarkers in a set of organisms” requires further investigation.

## 1. Introduction

Human population explosion has led to an era of rapid and heavy industrialization which threatens life in almost all possible habitats. The environment is being increasingly polluted with the addition of a large number of heavy metals, chemicals, and radiation, which are dangerous not only for human but also for other living beings. In these circumstances biologists are playing a pivotal role in creating awareness regarding the effects of hazardous materials along other issues towards the restoration and conservation of a healthy environment. Therefore, one key area of focus is the use of biomarkers as indicators of biochemical change, providing an early warning of environmental risk and its assessment. As such, biochemical markers often parallel changes in the physiochemical characteristics of the environment; the use of biomarkers as a detective measure can enable adopting some timely preventive measures to avoid certain hazards. To achieve these objectives, certain investigations have suggested the linking of stress genes, such as HSP70 and metallothionein, with a reporter gene for farmed vegetation that could be employed to enable the use of satellite images to gauge crop status for environmental health. Furthermore, these biomarkers may be utilized as tools to gain better understanding of the links existing among the environmental quality, food chains, and human health [1].

Until present, various biomarkers proposed for environmental risk assessment have been presented in Table 1.

The biomarkers presented in Table 1 have been considered as important tools for the identification and quantification of exposure, effects, or susceptibility in individuals of a population under adverse conditions. Therefore, biomarker selection should be primarily based on the ability of a biomarker being sufficiently sensitive to provide an accurate measurement in a sample.

Heat shock proteins (HSPs) are specifically produced when cells are exposed for a while to temperatures higher than their normal growth requirement. The synthesis of HSPs is a universal phenomenon in all studied plant and animal species including humans. Because HSPs can also be induced by oxidants, toxins, heavy metals, free radicals, viruses, and other stressors, they are sometimes called the “stress proteins” [2]. Heat shock proteins were initially discovered in 1962 in* Drosophila melanogaster* larvae in response to heat shock [3], and the term “heat shock protein” was coined by Tissieres et al. [4]. Heat shock proteins show characteristically marked variations in their expression in most of organisms under a range of temperatures [5, 6], heavy metals [7–10], chemicals [11–14], radiations [15–19], metabolites and hormones [20–24], clinical situations [16, 25–30] or pathogens that may be above or beyond their optimal limits [31–34], and drugs [35–37].

Heat shock proteins are cosmopolitan in all living organisms and are usually classified as different families according to their molecular size—HSP27, HSP47, HSP60, HSP70, HSP90, and HSP110. These families of heat shock proteins play crucial roles in physiological processes such as protein chaperoning activity, protection against apoptosis, steroidogenesis, and stress tolerance. In addition, heat shock proteins, especially HSP70 and HSP60, have also been proposed as biomarkers of exposure levels and toxicity. Their candidature as biomarkers is based on many observations showing that the HSP60 induction in certain organisms (e.g., mussel* Mytilus edulis* and nematode* Plectus acuminatus*) results in a several fold sensitive response than the use of other comparable parameters such as quantifying adverse effects on biomass or reproduction [38, 39]. Such an observation in mussels (*M. edulis*) led to their recommendation as one of the most suitable organisms for biomonitoring of aquatic ecosystems.

The role of HSP70/HSP60 as a biomarker is highly topical. Certain studies have produced contradictory results; some indicate a high HSP70 sensitivity to pollutants, while others suggest otherwise. The confounding issue in many such studies seems to be the use of varying concentrations of toxicity exposure; some authors have realistically used low concentrations, some have used high concentrations, but only very few tested real-world contamination exposures models. Furthermore, the existing validated bioassays, mostly based on lethality or reproduction, have somewhat limited applicability due to their crude sensitivity, long exposure, or the overall expenses of the test. In contrast, changes at biochemical level are usually the first detectable response to environmental disturbance. Therefore, analysis of toxicity-induced changes in gene expression (i.e., alterations in patterns of protein synthesis) and resultant cell injury may be quite handy factors to be considered as biomarkers of toxicity exposure. As these changes underline all effects at higher organizational level, therefore these have been regarded as highly sensitive indicators of toxicity.

## 2. Favoring Evidences: HSPs as a Biomarker

### 2.1. Aquatic Environment

The first favorable report on the use of HSPs as a toxicity biomarker came from the study of Cochrane et al. [40]. It was reported that exposure of rotifer* Brachionus plicatilis* to sublethal doses of CuSO_4_ resulted in a 4-5 fold increase in HSP58, with the maximum increase occurring at approximately 5% of the LC50 for the species. A similar response was seen with tributyltin. Kinetics of induction was sigmoid with induction occurring in the range of 20–30 *μ*g/L. However, no response was observed when exposed to Al, Hg, Zn, sodium arsenite, sodium azide, sodium dodecyl sulfate, or pentachlorophenol. It was suggested that HSP58 abundance might act as a biomarker of toxicity exposure.

A number of studies conducted on mollusks, mostly on mussels, for example, Sanders and Martin [41], reported elevated levels of HSP60 and HSP70 in mussels and fish tissue collected from polluted areas. The collection of data on sediment and water chemistry from the sampling sites and on contaminant body burdens indicated their exposure to contaminants was long-term. This study suggested that HSP accumulation might provide a method of quantifying adverse biological impacts of exposure to toxicants when examining wild populations from contaminated sites. Similarly, another study conducted by Porte et al. [42] illustrated statistically insignificant differences in the total levels of cytochrome P450 and benzo(a) pyrene hydroxylase activity and significantly induced HSP70 which correlated with the quantities of the PAHs accumulated in mussels* Mytilus galloprovincialis* (collected from sites polluted with aliphatic and polycyclic aromatic hydrocarbons—PAH). Again, it was suggested that HSP70 could be used as a biomarker in* Mytilus galloprovincialis* against PAH toxicity. Another study in mollusks considered HSP70 as biomarkers (in gill, mantle, and digestive gland) and the reason of survival of* Macoma nasuta* (clam) in response to heavy metals (Ni, Cr, and Cu) and trace organic pollutants (like PAH and organochlorine pesticides—aldrin and DDT and its metabolites DDD and DDE) exposure. Pearson and Spearman correlation analysis revealed that mortality and HSP70 in gills were significantly correlated with tissue concentrations of DDT and/or its metabolites [43].

Schröder et al. [44] validated hepatic HSP70 as a potential biomarker of cellular stress responses in fish* Limanda limanda* at spawning stage 2. Varying levels of HSP70 (consisting of two forms 75 and 73 kDa) among individuals were observed at different locations of North Sea (Germany), but each was correlated with intensity of DNA damage (single-strand breaks and alkaline labile sites). It was suggested that* L. limanda* might serve as a useful bioindicator and heat-shock proteins as a useful biomarker for the monitoring of environmental pollution. The fish epidermis is highly susceptible, as it is the interface between the fish and its surrounding aquatic environment. Heresztyn and Nicholson [45] investigated the use of HSP70 as a measure of sublethal ecotoxicity in cultured skin epidermal cells of rainbow trout* Oncorhynchus mykiss* exposed to chemical stress (2,4-dichloroaniline). A positive toxicant concentration-dependent increase was noted in the concentrations of HSP70 (quantified by immunocytochemistry). In addition to skin epidermal cells, the erythrocytes of certain fishes (e.g., silver sea bream* Sparus sarba*) have the ability to synthesize HSPs due to presence of a nucleus and therefore are considered as an interesting cellular model for in vitro toxicological studies. In one such study, their exposure to sublethal concentrations of Cd, Pb, or Cr-VI as low as 0.1 *μ*M (a value which represented threshold concentration in vitro) for 1 to 2 hours has shown significant overexpression of HSP70 [46]. Guizani et al. [47] also provided favoring evidence in support of HSP70 to act as biomarkers of environmental stress.

In addition to rotifers, mollusks, and fishes, some authors explored the potential of HSP70 as a biomarker of stress in algae and other plants. For example, Bierkens et al. [48] suggested that HSP70 in alga* R. subcapitata* is a sensitive biomarker, as it displayed a dose-dependent increase in response to a wide range of pollutants (ZnCl_2_, SeO_2_, lindane, carbaryl, and SDS—but not pentachlorophenol) at concentrations below the range of classical cytotoxicity (i.e., growth inhibition, lethality). However, ZnCl_2_ and SeO_2_ were found to be the strongest inducers of HSP70. In another study, Ireland et al. [49] reported elevated concentrations of HSP70 in toothed wrack* Fucus serratus* and common duckweed* Lemna minor* exposed for 24 hours to osmotic and cadmium stresses. In both stresses, the production of HSP70 increased to the maximum and subsequently decreased as the stressor levels increased. They suggested that HSP70, tested by an indirect competitive enzyme-linked immunosorbent assay, could potentially be applied for the stress detection in these aquatic species. In addition to HSP70 the biomarker response of MitosHSP to heat, ChlsHSP to H_2_O_2_, and antioxidant enzymes (Mn-SOD and Fe-SOD) and HSP60 to heat, H_2_O_2_, and Pb in the dinoflagellate* Karenia brevis* has also been reported by Miller-Morey and Van Dolah [50].

### 2.2. Terrestrial Environment

Various studies have also supported the use of HSPs as biomarkers in monitoring of soil pollution. In this context, most attention has been paid to soil invertebrates, especially* Plectus acuminatus* (nematodes),* Lumbricus terrestris* (annelids), and gastropods (mollusks) with the emphasis on HSP70 and/or HSP60 as a biomarker of toxicity. For example, Kammenga et al. [39] reported the induction of HSP60 related to increased concentrations of Cu and Cd (4–400 *μ*g/L and 7–700 *μ*g/L, resp.). For copper, the induction of HSP60 was three orders of magnitude more sensitive than was the EC_20_ for reproduction. For cadmium, HSP60 induction was one order of magnitude more sensitive. Their results pointed out that HSP60 induction occurred at concentrations that were realistic for the field situation (2 to 4.8 *μ*g of Cu for loamy sand and clayey soil). Therefore, HSP60 was suggested to be suitable as a potential biomarker to toxicant stress in* P. acuminatus*. About two years later, they presented a review on the potential and limitations of invertebrate biomarkers (including HSPs, metallothioneins and metal-binding proteins, esterases, histological and ultrastructural markers, lysosomal integrity, and the novel biomarker histidine) for soil risk assessment purposes. They concluded that the HSP response in soil invertebrates was especially suitable to indicate the effects of exposure to comparatively low concentrations for a range of toxicants and could be regarded as a biomarker of general stress [51]. However, in another study they suggested that HSP60 response in the nematode alone was not a suitable biomarker for heavily contaminated soils. It had indicative value related to the HSP70 response in the isopods (*Oniscus asellus and Porcellio scaber*) and could be a suitable biomarker for moderately contaminated soils. Furthermore, HSP70 concentrations in nontolerant individuals of both these isopods species were considered to be suitable for use as potential biomarkers for monitoring environmental pollution [52].

Nadeau et al. [53] reported that HSP70i analysis by western blot in intestinal tissues of* L. terrestris* was a suitable and sensitive bioassay for the assessment of adverse effects in earthworms when exposed to chemicals and heavy metals (chloroacetamide, pentachlorophenol, Pb, Cd, Cu, and Hg for 1–16 days). Their data also showed a good level of reproducibility despite some individual variations. In addition, they suggested that the use of animals from pristine habitats transposed into contaminated environments is of high ecological relevance. Induction of HSP70 in earthworms should represent not only a good wide-spectrum biomarker of exposure but also a biomarker of toxicity effects since known toxicants altered gene expression in these animals. Data gathered, therefore, is not simply a measure of accumulation of HSP. The detection of HSP70 in earthworms could represent an early-warning system for the presence of potentially deleterious agents in soils, particularly in* L. terrestris* and earthworms in general acting as potential sentinel animal species. In other studies, HSP70 has also been suggested as a sensitive biomarker in coelomocytes of the earthworm* Eisenia fetida* exposed to metals like Zn, Cu, Pb, and Cd (at concentration of 1.32 *μ*g/cm^2^ of filter paper) [54] and in McCoy cells exposed to low Hg, Cd, and CuCl_2_ concentrations (0.7, 1, and 3 *μ*M, resp.) [55].

### 2.3. HSP in Transgenic Systems

Some authors have emphasized the use of HSPs in transgenic cells and organisms for environmental risk assessment. Examples include transfected HeLa cells with firefly luciferase having the HSP22 promoter sequence of* Drosophila melanogaster* for CdCl_2_, Cd (NO_3_)_2_, NaAsO_2_, alachlor, fentin acetate, thiram, and maneb in the concentration range of 0.05–50 *μ*M [56]. Other examples include the use of transgenic* Drosophila melanogaster* (HSP70-lacZ) for the phthalimide group of chemicals, captan, captafol, and folpet [57], for cypermethrin (0.002, 0.2, 0.5, and 50.0 ppm), and for effluents of chrome plating industry containing Cr, Zn, Fe, Ni, Mn, Pb, and Cu [58, 59]. Embryos of a stable transgenic zebrafish with HSP70/eGFP reporter gene system, in which HSP70 expression was activated in a tissue-specific manner following exposure to a number of different toxins including Cd (data not given by author), served as a reliable and extremely quick indicator of cell-specific toxicity [60]. In transgenic zebrafish, reporter gene under human HSP70 promoter showed sensitivity to detect CuSO_4_ at doses as 1.2 m*μ*M [61].

Thus, HSP70 has often been highlighted as a particularly sensitive biomarker of exposure to different pollutants in earthworms, mussels, clams,* Drosophila*, certain fishes, algae, and some aquatic plants. Quite a few studies have suggested it as a biomarker of other adverse effects. Widely accepted models, such as* Drosophila* made transgenic for different stress genes, namely,* HSP70*,* HSP83,* and* HSP26,* tagged with reporter genes like *β*-galactosidase or GFP have been used to detect cellular stress caused by environmental chemicals or their mixtures [62]. The designed assays allowed the quantification of stress gene expression following chemical pollution exposure, suggesting the magnitude of cellular toxicity inflicted by the chemicals [63–68]. Nisamedtinov et al. [69] studied the response of the yeast* Saccharomyces cerevisiae* to different stress conditions employing transgenic technology. The group used HSP12p-Gfp2p fusion protein construct and demonstrated that the abundance of HSP12p under different environmental conditions depended on the specific stress factor. A rapid shift in stress factors gave higher rates of HSP12p synthesis compared to gradually changing stress conditions. Similarly, for developmental toxicity studies, zebrafish transgenic for* HSP70* or* HSP27* tagged with a GFP reporter were exposed to heavy metals to examine the effect of the xenobiotics on different stages of development [70].

## 3. Contradictory Evidences

A number of researchers have criticized the use of HSP70 or HSP60 as biomarkers. Wieganta et al. [71], for example, reported that different stressors (arsenite, cadmium, dinitrophenol, and ethanol) recognized as well-known HSP-inducers, failed to stimulate specific HSPs in rat hepatoma cells to a degree that is comparable to the induction of these HSPs by thermal shock. Therefore, validation of stressor-specific risk assessment was considered through further research with larger groups of proteins. Mirkes et al. [72] reported that the heat shock response, characterized by the synthesis and accumulation of HSP72, was not a general biomarker in rat embryos for chemical teratogens such as N-acetoxy-2-acetylaminofluorene, CdCl_2_, cyclophosphamide, sodium arsenite (AS), and sodium salicylate (SAL). Last two chemicals induced the synthesis and accumulation of HSP72, and both have different accumulation kinetics; otherwise, these chemicals caused embryotoxicity characterized by abnormal development and growth retardation. Overexpression of HSP72 after short-term exposure (2–6 hr) of pulmonary cell line (A549) to acute Cd concentrations (higher than 50 *μ*M) was considered an early biomarker for occupational exposure to Cd but long-term (1 month) chronic exposure* in vivo* made it doubtful because the expression of HSP72 decreased due to cellular adaptation to chronic Cd exposure [73]. Similarly in juvenile rainbow trout exposed to Cd (1.5 *μ*g/L) and Zn (150 *μ*g/L) for 21 days, an adaptive response, to a lesser extent, in the liver was shown by an increase in antioxidant defenses (total glutathione, superoxide dismutase, and Trolox equivalent antioxidant capacity) without any impairment of GSH redox status or induction of HSP70 and HSP60 [74].

Efremova et al. [75] reported that Pb and Zn caused a strong induction of HSP; organochlorines and pentachlorophenol also caused induction but did not enhance consistently. Wastewater from the Pulp and Paper Plant caused a concentration-dependent increase in HSP70 expression in freshwater sponges endemic in Lake Baikal. However, there was no difference in the basal concentrations of HSP70 between sponges collected from polluted (with wastewater of Pulp and Paper Plant) and unpolluted sites. Another study on the sponge* Crambe crambe* reported that the accumulation and response of HSP54 were higher and quicker than HSP72 for Cu exposure. However, HSP72 was significantly induced only in the individuals transplanted to the contaminated site. Under experimental conditions, both heat shock proteins were induced by copper at 30 *μ*g/L and inhibited at 100 *μ*g/L. The highest mean values of HSP54 and HSP72 corresponded to the sponges that showed the lowest mean values of toxicity. Thus, toxicity and production of HSP displayed antagonistic trends [76]. In ascidian* Pseudodistoma crucigaster,* HSPs were induced only where Cu concentrations were under half of the actual concentration in their polluted harbor. The author suggested that HSP was useful only as an early warning system for sublethal Cu pollution in the ascidian, as the response was inhibited above a threshold value of the stressing agent, which was variable among species [77]. Furthermore, the effect of Cu (50, 100, and 960 *μ*g/L for 2–24 h at 19°C) on the levels of HSP60 analyzed by western blotting analysis of the planarians* Dugesia schubarti* revealed no changes in HSP60 expression. However, catalase activity was significantly affected. Therefore, it was concluded that HSP60 should not be used as biomarker for Cu pollution in planarians [78].

Insect* Tetrix tenuicornis* was collected from polluted and unpolluted sites for studying heavy metals accumulation and its impact on stress proteins level. Insects collected from a polluted area had higher concentrations (ranging from 1.5- to 42-fold) of Cu, Zn, Pb, and Cd than control insects. Moreover, heavy metals accumulations caused only minor variations in the accumulation of Hsc70 and HSP70i [9]. Furthermore, an inhibition of HSP70 synthesis has also been observed in the earthworm* Lumbricus terrestris* in response to a variety of metals such as Pb, Cd, and Cu [53]. While evaluating the power of HSP70 as an environmental biomarker of fish health during field conditions, Webb and Gagnon [79] reported that HSP70 measurement alone was insufficient to evaluate fish health conditions.

## 4. Discussion: Critical Analysis

A general conclusion regarding the validity of HSP70 and/or HSP60 as biomarkers of toxicity exposure or effect is difficult to reach, in part due to the conflicting nature of many of the above research reports. However, certain research reports enable us to draw conclusions regarding the strength of specific biomarker candidates. In analyzing the research to date, we have encountered 9 basic concerns regarding the limitations of the use of HSP as biomarkers. These are explained in detail in the following discussion. The following concerns should be addressed by the researchers in order to reach some concrete conclusions.

1. Can these HSPs as biomarkers reveal both the concentration and nature of a specific toxicant in an environment?
2. Are uptake, threshold concentration, and inducing ability of all metals/toxicants the same?
3. Do variability of HSP70 concentrations among various organisms and the acquisition of tolerance significantly affect the results?
4. Do age and gender based differences have no effect on expression of HSP70?
5. Are physical properties of soil and variable detoxification mechanisms among species not acting as confounding factors?
6. Does a synergistic effect of different toxicants along environmental factors modify the expression of heat shock proteins?
7. Do different techniques for HSP detection produce similar results for a particular toxicant in the same organism?
8. Are the studies validating HSP as biomarkers broad enough?
9. Is application of biomarker sets a useful strategy for assessment of toxicity?

### 4.1. Concentration and Nature of a Specific Toxicant

These HSPs did not reveal the concentration or the nature of a specific stressor, that is, type of heavy metal or chemical, as these showed a marked variation in their concentrations in response to a variety of stressed conditions. Moreover, these concentrations did not give a true picture of different toxicants in an environment, that is, how many toxicants were present in a specific locality or habitat under investigation.

### 4.2. Uptake, Threshold Concentration, and Induction

Threshold metal concentrations for the HSP70 induction vary among different metals. For example, in zebra mussels (*Dreissena polymorpha*) metal uptake analysis for Pt, Pd, Rh, Cd, and Pb indicated the highest uptake for Cd followed by Pt, Pb, Pd, and Rh. The highest HSP70 values were observed in the case of exposure to Pd followed by Pt, Rh, Pb, and Cd. Therefore, Pd seems to be a particularly potent inducer of HSP70, despite its relatively low threshold concentration [8]. Therefore, simple conclusions on the basis of body burdens should be avoided and results should be interpreted on the basis of strength of toxicant. Furthermore, the potency of various toxicants significantly differs in inducing HSP70 promoter. For example metals like Cd, Zn, and Hg and organic compounds like chlorophenol derivatives, 3, 4-dichloroaniline, ethyl parathion, benzo(a)pyrene, 2, 4-dichlorophenoxyacetic acid, endosulfan, diuron, and 4-nonylphenol are effective at noncytotoxic doses, while tetrachlorohydroquinone and 1-chloro-2,4-dinitrobenzene induce the promoter at cytotoxic doses [13]. Occasionally, one metal enhances the accumulation of others. This is the case with a combination of Cu and Zn which resulted in higher accumulation of Zn in HepG2 cells [80]. Occasions were also noted where HSP70 concentrations did not correlate with the degree of metal exposure (as in crabs* Carcinus maenas* for Cu and Zn) [81]. Furthermore, a deficiency of certain metals, for example, Cu, reduced the expression of HSP70 in certain tissues (cardiac) and their mitochondria [82], and the exact effect of such dietary deficiency on other organs/cases is still in question.

### 4.3. Interindividual Variability of HSP70 Concentrations and Acquisition of Tolerance

Individual differences exist even at molecular levels, a factor, for instance, giving rise to individual variability in HSP70 expression in intestinal tissues of* L. terrestris* [82]. Some authors have reported a weak correlation between toxicant and HSP70 as biomarkers due to interindividual variability of HSP70 concentrations. This was the case in the bay mussel* Mytilus trossulus,* where the interindividual variability tended to mask inductions of HSP70 at low concentrations of As(III), thus making it a less efficient biomarker of toxicity. To avoid such problems pre- or poststress specimens are required to be analyzed to allow for greater HSP70 sensitivity and reliability. Other markers, such as the use of actin, are required to be used as controls; the use of HSP70-reporter gene constructs is suggested and detection with HSP60, heme oxygenase-1, metallothionein, CYP450, MXR, or GPx has been recommended by La Porte [83].

Another difficulty in validating HSPs as biomarker for ERA is the enhancement of stress response (in form of increased synthesis of HSP70/HSP60) in self-tolerance as well as in cross-tolerance by nonlethal doses of toxicant in sensitized cells/tissues (the cells or tissues already exposed to such toxicants in self-tolerance or to other toxicants in cross-tolerance). An example is amphetamine and Cd in hepatic cells [84, 85]; Hg [86] and uranium [87–89] in the kidney are able to induce self-tolerance, while Zn against Cd in rat proximal tubule cells [90], Pb against Cd/C_2_H_2_ in astroglial cells [91], As, Cd, Hg, Pb, Cu, menadione, and diethyldithiocarbamate against lethal temperature in Reuber H35 hepatoma cells [92] were also able to induce cross-tolerance. “How many times?” and “To what extent has an organism been exposed?” are questions to be addressed under the natural environments. Therefore, acquired resistance against cytotoxicity as well as cross-tolerance in a large number of organisms also adds uncertainty in the role of HSP as biomarker in ERA. Likewise, biomarker responses vary in relation to the duration and level of exposure under laboratory conditions and are also dependent on the population [93, 94]. Acquisition of tolerance by certain organisms not only masks the true picture but also seems as a dubious factor when applying the issue to the establishment of water quality criteria [95].

### 4.4. Age and Gender Based Differences

Certain studies have revealed that not merely the functional ability of HSP70 against stress conditions that decreases with aging [96] but also aging is associated with an actual decreased concentration of HSP70 and subsequently a decreased ability to respond to stressed conditions [97]. Certain studies have also revealed gender based differences regarding expression of HSPs. For example, HSP70 gene assay in the reproductive organs of adult flies showed its expression restricted to male flies [59]. Considering these studies, such gender and age based differences are also expected in other organisms with respect to HSP70 in response to toxicants. The age and sex of an organism should also be considered, especially when studying the role of HSPs as a risk assessment of environmental pollution's effects in sex organs.

### 4.5. Confounding Factors

In case of soil pollution, Filzek et al. [98] emphasized the consideration of the underlying geology, the nature of the soil, and the land use as essential prerequisites to understand the significance of any observed biological effects. The authors also provided extensive discussion on how the availability and mobility of various heavy metals at the selected field sites were influenced by a wide range of factors such as pH, organic matter, and clay content. The significant differences in toxic responses measured in the laboratory exposed versus field exposed nematodes by Arts et al. [52] were explained due to confounding factors such as food availability and differences in contaminant uptake routes under the different exposure regimes. It was also suggested that differences existed between native animals transplanted to the field and field collected animals, partly attributable to the increased and probably inherited tolerance of the field population. Furthermore, physiological differences in the way an individual species handles the uptake, detoxification, assimilation, and eventual excretion of accumulated metal also influenced the HSP70 response in isopods. Such physiological differences exist not only in terrestrial organisms but also in aquatic [99].

### 4.6. Synergistic Effects among Toxicants as well as between Toxicant and Environmental Factors

**Figure 1:**
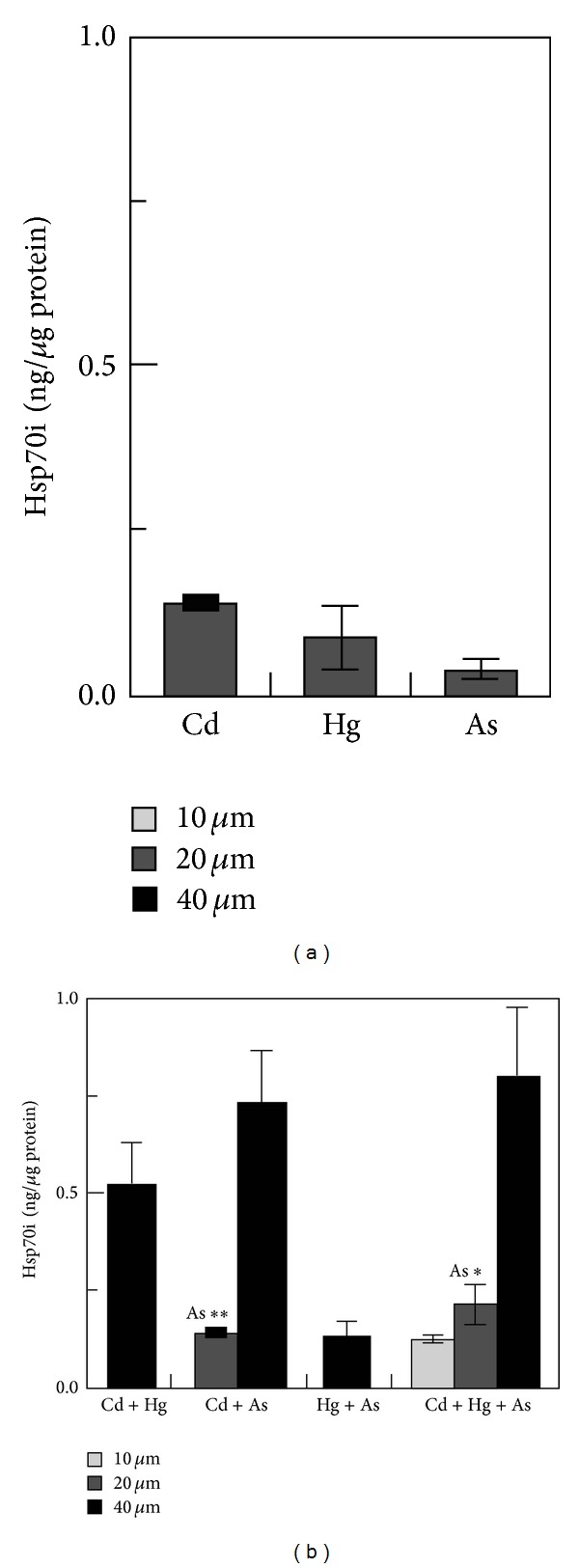
Toxic metals induce HSP70i accumulation in podocytes in a dose-dependent manner. Results of a quantitative western blot analysis of HSP70i accumulation in podocytes treated with various concentrations of individual toxic metals (a) or combinations of two and three toxic metals totaling 10, 20, or 40 mM (b) for 3 days. Values are expressed as ng of HSP70i per mg total protein. Basal HSP70i levels were below the limits of detection [100].

**Figure 2:**
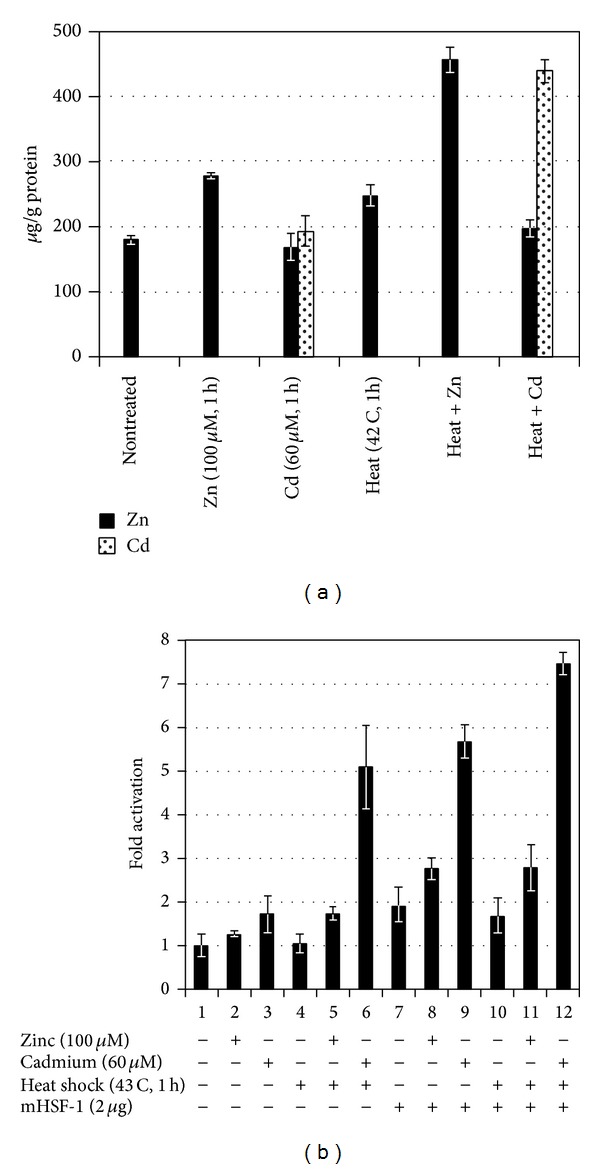
(a) Cellular accumulation of Zn and Cd is boosted by heat shock. After addition of Zn and Cd to final concentration of 100 and 60 uM, respectively, with or without heat shock (42°C for 1 h). HEK293 cells were harvested and analyzed by ICP-MS. The data from three independent determinations has been shown [101]. (b) Expression of HSP70 promoter by Cd and heat in presence or absence of HSF-1. HEK293 cells were transfected with HSP70-Luc promoter-reporter construct, the CMV-LacZ reference construct, and mouse HSF-1 expression vector. 36 h after transfection, cells were treated with 100 *μ*M ZnCl_2_ or 60 *μ*M CdCl_2_ with or without heat shock at 43°C for 1 h. The cells were collected and reporter gene activities were determined by luciferase assay. The basal level was taken as 1 to calculate the fold activation [101].

**Figure 3:**
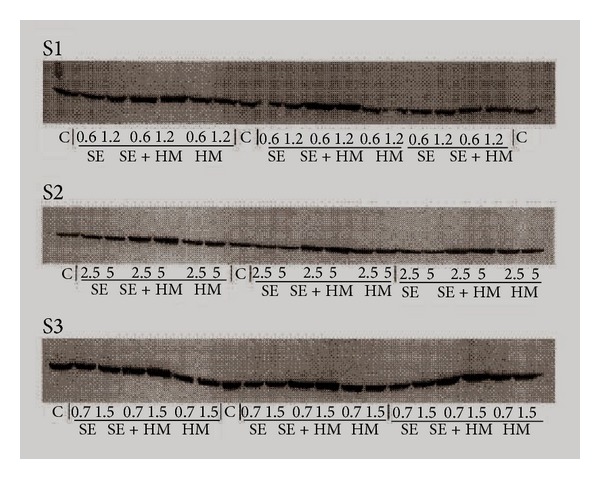
Immunoblots showing HSP72 levels in HT29 cells after a 24 h exposure to the different sludge extracts at different concentrations. Cells were subjected to soluble sludge extracts alone (SE), to soluble sludge extracts + heavy metal solution (SE + HM), or to heavy metal solution alone (HM) at the indicated concentrations expressed as grams per kilogram of dry material. Untreated cells are control (C) S1, S2, S3, different sludge [102].

**Figure 4:**
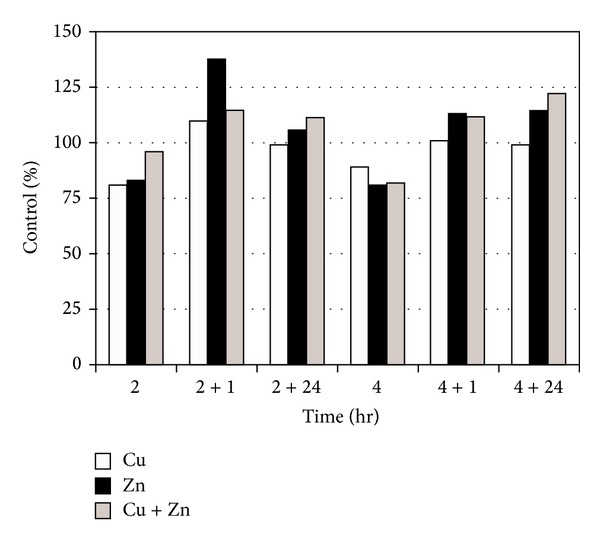
Scanning densitometry analysis of HSP70 in Cu (30 *μ*g/mL) and Zn (50 *μ*g/mL) alone or in combination treated HepG2 cells expressed as percent of control at the time described [80].

No data exist regarding the synergistic effects of different toxicants and/or with the other aspects of environmental stresses (temperature, pH, salinity, etc.). For instance, the toxic effect is significantly altered under the additive effects of many heavy metals as compared to cases of isolated single metal toxicity where two or more metals are found in a combination. Individual dose of 20 *μ*M of As, Cd, and Hg induced only a modest HSP70 increase, whereas their combination at the lowest levels of toxicity still induced a greater accumulation of these proteins (Figure 1) [100]. Furthermore, a number of stress genes which respond to heavy metals (such as HSPs and MT) contain metal-response elements (MREs) in their promoter/enhancer region, which is activated by a metal-responsive transcription factor-1 (MTF-1). The response to heat shock is mediated by heat shock transcription factor-1 (HSF-1), which activates a battery of heat shock genes. Synergistic activation has also demonstrated the metal-responsive promoters by heavy metals (Zn or Cd) and heat shock in combination. Heat also stimulates the intracellular accumulation of Zn and Cd when provided exogenously during a heat shock, (in HEK293-mammalian cells) and thus results in a hyperactivation of the metal response pathway. Interestingly, relatively low concentrations of these heavy metals alone hardly induced transcription at all and served as sufficient trigger for such synergistic activation of mammalian HSP70 promoter (Figures 2(a) and 2(b)) [101]. Similarly, water-soluble fractions of different sludge containing varying concentrations of heavy metals (Cd, Cr, Cu, Ni, Pb, and Zn), when given separately to human cultured cells (HT29 cell line from gut mucosa), failed to trigger significant expression of HSP72. When given in combination, they exerted a strong synergistic effect by causing significant overexpression of HSP72 (Figure 3) [102]. Increasing concentrations of HSP70 have also been observed in HepG2 cells under the synergistic effects of Cu and Zn as compared with each metal (Figure 4) [80]. Another study conducted by Aït-Aïssa et al. [74] reported that 3,3′,4,4′-tetrachlorobiphenyl (1 mg/kg) strongly induced HSP70, while its coexposure with metals did not modulate significantly its effects. However, 17-beta-oestradiol in combination with Cd/Zn had shown a synergistic effect.

**Figure 5:**
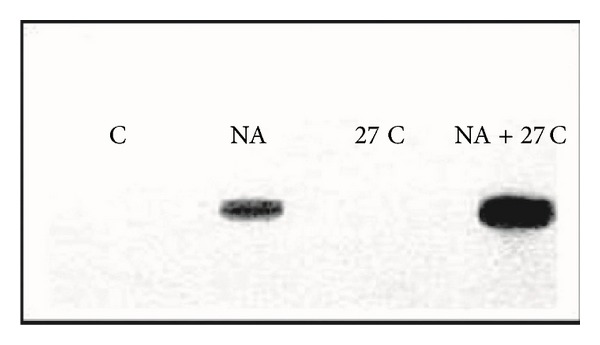
Effect of 2 h heat shock (27°C) and sodium arsenate (10 *μ*m) in A6 cells of* Xenopus laevis* with control 22°C [120].

**Figure 6:**
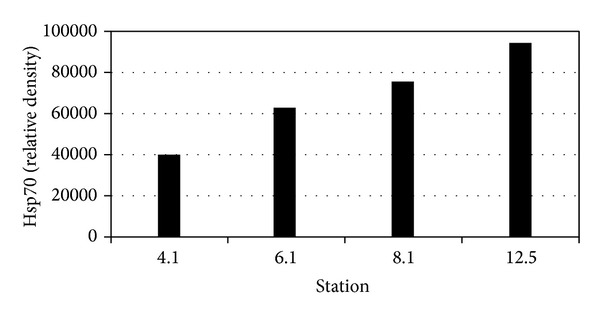
HSP70 levels in* Potamocorbula amurensis* (means ± SD, *n* = 90) measured each month (*n* = 6–8 per station) from 07/1996 to 01/1998 (except 10/96, 1–3/97, and 10/97). One-way ANOVA and Tukey analysis revealed two major groups of sites: group A with sites 4.1 and 6.1 and group B with sites 8.1 and 12.5 (*P* < 0.001) [106].

**Figure 7:**
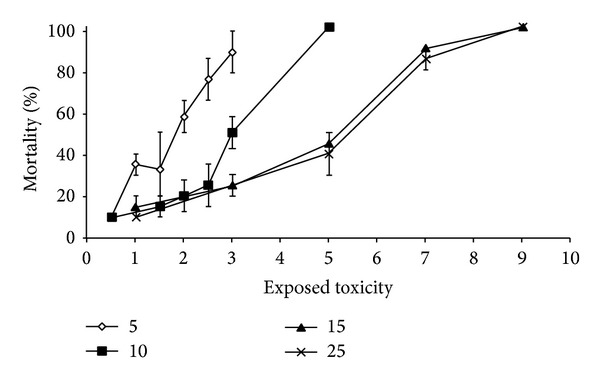
The concentration-response curve of* Neomysis integer* for a mixture of six metals at different salinities (5, 10, 15, and 25‰). The toxicity decreases with increasing salinity and higher salinities above 25‰ had no further influence on the 96 h LC50 of the mixture of six metals (Hg, /Cd, /Cu, /Zn, Ni, /Pb) which is situated at a value between 4.4 and 4.6 T.U [104].

Apart from the synergistic effects of toxicants, environmental factors such as temperature (Figure 5) [103], salinity [104], and oxygen supply [105] also influence the expression of HSPs and may also have a synergistic effect in combination with toxicants, thus making their consideration as biomarkers doubtful. For example, Cd exposure increased the HSP70 concentrations in marine clams, whereas salinity markedly lowered the same level in that species. A laboratory study regarding the effects of salinity on HSP70 concentrations indicated that exposure to 0.1 ppt salinity markedly lowered HSP70 concentrations in clams* Potamocorbula amurensis* compared with those exposed to higher salinities (Figure 6 and Table 2) [106]. Increasing the salinity from 5 to 25 per thousand resulted in lowering the toxicity and concentrations of the free metal ions (Figure 7). This effect has been regarded as the strongest for Cd and Pb, while such smaller effects were observed for Ni, Cu, and Zn [104]. The rate of uranium accumulation in the gill tissues of clam* Corbicula fluminea* was higher under hypoxia than normoxia. At the cellular level uranium instead of hypoxia induced the expression of multixenobiotic resistance protein. On the contrary, HSP60 was induced by hypoxia instead of uranium [107].

Some authors have also regarded HSP70 as a biomarker in the algae* Raphidocelis subcapitata* in response to changes in pH, temperature, humic acids, nitrates, and phosphates. Algae responded to these changes by a transient increase in HSP70 concentration. Temperature and pH were found to induce acquired tolerance; that is, algae grown at a pH or at a temperature different from control conditions were shown to have acquired resistance to a subsequent challenge with Zn (10^5^ M). These results qualify HSP70 as a biomonitor for environmental pollution provided that essential environmental parameters such as pH and temperature are kept constant [108]. Apart from temperature and pH, much similar emphasis has also been placed on the analysis of nutrients, electrolytes, and dissolved oxygen content [105].

Another environmental factor influencing the HSP expression is seasonal variation, which acts partially according to the corresponding temperature regimes. Seasonal variations in HSP70 as noted in the mussel (*Mytilus galloprovincialis*) at two sites of Mediterranean Sea Carteau (native site) and La Fourcade (transplantation site) in a two-year study may be more likely a result of combined environmental factors (temperature, salinity, and turbidity) and chemical contamination levels [109]. Bodin et al. [109] conducted a comprehensive study; they did not suggest any correlation of variations in biomarkers level with other parameters. They also reported that mussels of both sites have specific chemical contamination profiles but having a similar range of values. For example, both sites were highly contaminated by heavy metals (201 and 258.4 mg/kg dw, resp.) and considered as moderately impacted for polychlorinated biphenyls and polycyclic aromatic hydrocarbons; nevertheless, contamination levels at Carteau were twice as high for PAHs (101.5 mg/g dw) and PCBs (90.2 mg/g dw) as La Fourcade. The seasonal contamination trend at Carteau showed a sixfold higher level of pyrolytic pollutants in winter. It showed that seasonal variation in contamination levels was a man-made activity which correlated well with their daily life needs [110]. Another study by Hamer et al. [111] investigated the concentrations of HSP70 in the gills of the mussel* Mytilus galloprovincialis* seasonally collected from different sites of the Rovinj coastal area (Croatia). They observed maximal levels of HSP72 and HSP70 in summer (September) and minimal concentrations in winter (December). HSP70 showed significant correlation with the sea temperature (*r* = +0.822, *P* < 0.05) only. Similarly, a significant seasonal (March and September) difference in HSP70 content has also been found in centipedes collected from unpolluted areas [112]. Female fishes collected from two different localities during spring (26.5°C) and winter (4.8°C) also displayed a similar trend in the ovarian and liver tissue of the black bullhead* Lepomis macrochirus,* in head and kidney of the bluegill sunfish* Ameiurus melas,* and in the gill tissue of both species [113].

Thus seasonal variations act through temperature and nutritional regimes, as well as through quantity and quality of pollutants dumped into environments according to seasonal activities of human beings. As a result, complex molecular interactions in actual environmental habitats are operating on biological structures, and in the case of chronic pollution the action of the toxic substances may not be predominant but is associated with many other environmental stressors. In combination with other environmental factors, pollutants can contribute to the weakening of defense and regulatory mechanisms of studied organisms. Hence, the biomarkers of exposure related to these mechanisms of early physiological regulation are subject to variations that make it difficult to detect the specific effects of chemical pollutants. The interference of natural environmental factors in the expression of biomarkers is an important issue with respect to the use of biomarkers in monitoring the biological effects of pollutants in their natural environments, making field interpretations difficult. Therefore, the effects of environmental factors should also be considered in sampling strategies for monitoring programs to prevent false interpretation of results. Furthermore, certain field studies have also shown that stress response can occur even at minute pollutant concentrations that are usually prevalent in the environment. Increasing knowledge on the kinetics and persistence of the stress response to complex environmental mixtures (the influence of both physiological and environmental parameters), the constitutive levels of HSPs and the acquisition of tolerance are required before safe application of HSPs to assess onsite pollution.

### 4.7. Detection of HSPs through Northern and Western Blot

Normally, both techniques are used for HSPs detection to quantify HSPs as biomarkers of toxicity. Some researchers emphasized that northern blot is a highly sensitive and initial step in the detection of environmental stress on gene expression. Other scientists emphasized on western blot by the fact that changes in mRNA expression do not necessarily correspond to changes in protein levels [7] or that polyribosome may be involved in protein synthesis under certain circumstances. A simple measure of mRNA may yield a doubtful reliability. Which is really more reliable remains questionable. For some HSPs, a correlation was noted between mRNA induction and its proteins (HSP60, HSP68, and HSP84) [114]. Some others did not find any correlation between mRNA levels and protein synthesis, for example, HSP68 [115]. Some cases have also been seen where mRNA levels remained constant after exposure to heavy metals (ZnCl_2_, 0–330 *μ*M), while protein levels significantly increased in a dose-dependent manner [116]. Hence, conclusions cannot be drawn only on the basis of either one, and both aspects should be explored in proposed model organisms.

We do not intend to imply a lack of quality of the work in the studies validating HSPs as biomarkers. In fact, all such studies are valid and we acknowledge them for their valuable findings. However, in our opinion, there are some logical questions that must be addressed with reference to certain studies. For example, Arts et al. [52] narrated that HSP60 response in the nematode* Plectus acuminatus* had an indicative value related to HSP70 response in isopods and could be a suitable biomarker for less heavily contaminated soils. Such reasoning is ambiguous. If we need to check the extent of soil contamination prior to concluding the significance of a biomarker response then what is the advantage of such biomarkers?

Most of the studies validated HSPs as biomarker of toxicity on the basis of 2–5 toxicants and even some on the basis of only a single toxicant [58, 59, 78]. Just a very few investigators considered synergistic effects. Aït-Aïssa et al. [13] investigated the maximum number of toxicants (3 metals and 15 organic chemicals) in this context. We were unable to locate any reference for the studies with analysis of significantly broad range of toxicants or studies containing all possible heavy metals or all organic pollutants. If an initial study used Cu, Cd, and Hg for gill and hepatopancreas in fishes, for example, a more useful continuation of such research would be to continue testing different metals on the same organs of same animal rather than using the same metals for a different animal. In this latter case it is very difficult if not impossible to validate HSPs as a biomarker of toxicity exposure or of adverse effects. For example, if we are testing the organism for environmental risk assessment in which expression of HSP70 or HSP60 was considered as biomarker in response to Cu, Cd, Zn, and Hg, the environment may also have Cr and/or organic toxicant in addition to electromagnetic waves; then how can we interpret the results, as HSPs respond to a variety of toxicants in suppressive as well as in overexpressive ways depending on the concentration of toxicant and duration of exposure? To broaden the testing in a range of potential toxicants and environmental factors for a single species would advance the field to a far greater extent.

### 4.8. Application of Multiple Biomarkers as a Set

The relationship of one biomarker to other potential biomarkers on exposure to particular contaminants in different species and in different organs or tissues of the same species is a key consideration prior to their widespread application in environmental management. Though more complex, such a technique may enable the use of biomarkers to give a much clearer picture of the environmental situation. Certain studies have already begun to consider this area. Their systematic approach is being considered here as follows.

- Certain studies have used various xenobiotics as a tool to study stress protein synthesis in target organs in order to evaluate the target tissue-specificity of the toxicant. For example, the kidney is a target tissue for chronic Cd exposure, so HSP expression in it can be used as biomarker [117] and even some metals like Hg induce regional and cell-specific stress protein expressions in rat kidneys [86]. Similarly, tissue-specific differences in the accumulation of HSP70 and HSP60 in* Mytilus edulis* exposed to a range of copper concentrations have also been reported by Sanders et al. [38]. Such studies could help in the selection of a combination of target tissues/organs for evaluation of HSPs as a biomarker for ERA.
- Some stressors have quite opposite effects on the expression of HSP70 in different cases. For example, Ni concentration of 600 M has no effect on HSP70 expression at the transcriptional level in HeLa cells [118], whereas it has shown sufficient expression in black sea bream fibroblast cell line at concentration of 0.01 M [10]. Arsenite is a much lesser inducer of MT but a more effective inducer of HSPs, while nickel is a good inducer of MT but poor inducer of HSPs [119]. These studies suggest the application of such biomarkers in combination as a set for ERA.
- Toxicity responses broadly vary among individuals of different species. Some examples exist where toxicity stress alters the HSP levels in some organs (e.g., in gills and livers in case of trout) and in some cases whole of the organism as in gammarids [105]. Such studies and those that have contradicted the validity of HSPs as biomarker for ERA can still help us in the selection of organisms.
- The expression pattern of HSPs is not only tissue-specific [34, 120] but also species-specific, as revealed by decrease, moderately increase, and overexpression of HSP73 in COS-7 cells (African green monkey kidney cells), A549 cells (human lung tumor cells), and rats kidney cells, in exposure to 100, 200, or 400 M NiCl_2_, respectively, for 4 days [121]. Such studies suggest the selection of organisms as a combination set for ERA.

To determine the variability of sublethal effects of pollutants, only a few studies have been conducted up to the third point. For example, Downs et al. [122, 123] developed a molecular biomarker system (MBS) based on 9 specific cellular parameters to assess the physiological status of the grass shrimp* Palaemonetes pugio* (exposed to Cd, atrazine, and bunker fuel) and mud snails* Ilyanassa obsoleta* (exposed to Cd, atrazine, bunker fuel, endosulfan, and heat stresses). They assayed HSP60, HSP70, alpha B-crystallin homologue, lipid peroxide, total glutathione level, ubiquitin, mitochondrial manganese superoxide dismutase, metallothionein, and cytochrome P-450 2E homologue. They reported that MBS was distinguishable among responses to each stressor and to nonstressed control conditions; that is, the biomarkers metallothionein and cytochrome P450 2E homologue distinguished between metal and nonmetal stresses. Aït-Aïssa et al. [74] confirmed by multivariate analyses that some correlation exists between these biomarkers and concluded the use of complementary biomarkers as necessary to discriminate between different treatments and to highlight interactive effects. In addition to animals, HSP70 could potentially be applied to the detection of stress in aquatic plants like* Fucus serratus* and* Lemna minor*. But it would be most effective when used in conjunction with other measurements to provide a stressor-specific biomarker profile or fingerprint [49].

In the case of soil, Arts et al. [52] reported that HSP60 response in the nematode* Plectus acuminatus* had an indicative value when related to HSP70 response in isopods and could be a suitable biomarker for less heavily contaminated soils. Similarly, for analysis of HSP70 in coelomocytes, Homa et al. [54] also reinforced the notion given by earlier studies that the value of biomarkers is higher when they are employed in combination as suite rather than individually [93, 124].

Previous researchers tested only a limited number of toxicants and, in some cases, only heavy metals were under investigation. The application of these suits is possible in an already tested environment/locality with similar levels of toxicants and conditions. In any other region with a different nature or extent of contamination results may remain confounded. However, such an approach can yield better results if applied like a taxonomic key.

Gupta et al. [62] considered HSPs as suitable early warning bioindicator of cellular hazard. It was further argued that despite having enormous use in toxicology, the current state of knowledge in defining a mechanism of action or accurately predicting toxicity based on stress gene expression warrants further investigation. The properties of heat shock proteins as (i) part of the cellular protective machinery, (ii) inducible nature against a wide range of chemicals, and (iii) higher conservation across the taxa have proven them to be valuable as a first tier biomarker in risk assessment. Although stress gene expression has proved promising to understand the toxicity of chemicals, literature linking activation of stress genes to mechanism of toxicity is limited [62].

In a recent study, HSP expression profile was used as biomarker in the fish for monitoring the water quality of a river [125]. Four of HSP genes, namely, HSP30, HSP60, HSP70, and HSP90, were amplified and sequenced by using degenerate primers. Later on, gene specific primers were developed and subsequently used to monitor the expression of above mentioned four genes. Compared to the fish at reference site, up to 10-fold difference in expression of HSP 70 was observed, in the liver. More profound differences were observed in expression of HSP30 in the kidneys of fish. However, as far as HSP60 and HSP90 are concerned, no difference in expression level was observed. These differences in HSP expression correlated well with the quality of water; more profound differences were observed in downstream water as compared to upstream. Authors were of the opinion that HSP30 and HSP70 expression can be used as biomarker for evaluation of water quality [125].

Transcription profiles of two of HSP genes, that is, HSP70 and HSP90, were used as biomarkers against metals and organic compounds' stress in marine diatom* Ditylum brightwellii* (Db) [126].* D. brightwellii* cultures were exposed to various metal compounds, namely, CuSO_4_, NiSO_4_, CuCl_2_, and NiCl_2_, and HSPs gene expression was monitored by using real-time PCR. It was observed that all tested metal compounds induced the expression of HSP90 gene; however induction pattern was different according to the tested metal compound. All concentrations of CuSO_4_ effectively induced the transcription of HSP90 while only higher concentrations of CuCl_2_ and NiSO_4_ were able to cause a significant increase in expression. NiCl_2_ initially increased the expression of HSP90 in a concentration-dependent manner. However, at much elevated level opposite effect was observed; that is, expression decreased with further increase in NiCl_2_ level. HSP70 expression followed a different pattern; expression was induced by CuSO_4_ and NiSO_4_ but not by CuCl_2_ and NiCl_2_. Moreover, the expression of former two genes was not concentration dependent. In the same study, effect of thermal stress and organic pollutants was also analyzed. Thermal stress induced expression of both genes; however, tested organic pollutants had no significant effect on expression of HSP genes. This data shows that HSPs are differentially involved in defense against various stressors. Based on findings of above mentioned study, an important point needs to be considered; it seems that anionic conjugates of metal (SO_4_, Cl_2_, etc.) may be responsible for specific induction of HSP genes. This can be observed from the fact that metal ions conjugated with SO_4_ were able to modulate the transcription of HSP70; however this was not the case for metals conjugated with Cl_2_ [126].

Transcription profile of HSP70 was also studied in* Mytilus coruscus* in response to fuel and heavy metals stress [127]. In all the treatments HSP expression was induced with varying degree of magnitude. HSP70 expression steadily increased with the passage of time and reached to the maximum (about 6-fold increase) after 25 days of the treatment. However, expression started decreasing after gaining the peak level, and at day 30, expression was about twofold of the control. Similar pattern was observed for heavy metals Cu^2+^ and Cd^2+^ but with more pronounced increase in the expression level of HSP70. Both Cu^2+^ and Cd^2+^ enhanced expression to 10-fold and 11-fold, respectively. However, time of maximum expression was different; in the case of Cu^2+^, peak expression was achieved at day 15 while in the case of Cd^2+^ it was at day 9 [127]. Although authors have advocated HSP70 as potential biomarker for heavy metals and fuels, this notion is questionable due to the fact that HSPs expression is responsive to other environmental factors like increase in temperature. Moreover, it seems, at least, in this study by Liu et al. [127] that HSPs expression showed a delayed response to heavy metals stress, as compared to hydrocarbons. In the latter case, expression was modulated in matter of hours while for heavy metals, it happened in days. In real environmental conditions it will become almost impossible to correlate the change in expression of HSPs to a particular stressor.

HSP70 expression was taken as one of the biomarkers for studying the effects of heavy metal accumulation in milk fish (*Chanos chanos*) collected from polluted sites of Kattupalli Island, India [128]. Through immune fluorescence scanning and western blotting, HSP70 accumulation was observed in gills and liver of the milk fish. Expression of HSP70 was more in the gills of fish collected from polluted site as compared to that from less polluted site. Similar expression pattern of HSP70 was observed in the liver tissue of the fish [128]. The authors have shown more realistic approach in drawing the conclusion based on this study. In spite of showing optimism in taking HSPs as a biomarker, they have stressed on more integrated approach for assessment of metal contamination in ecosystem. To summarize, HSP70 can be taken as biomarker, however not in isolation. Instead it could be more logical to use it as biomarker along with other parameters like oxidative stress biomarkers and ultrastructural changes.

Few reports have also shown the downregulation of HSP70 gene in response to heavy metal stress. In a recent study, Luo et al. [129] have observed the effect of long-term heavy metals stress on* Crassostrea hongkongensis*, through a proteomic approach. Differentially expressed proteins were identified in oyster exposed to heavy metals such as zinc, copper, manganese, and lead. One of differentially regulated proteins was identified to be HSP70. In contrast to usually reported upregulation, HSP70 was found to be downregulated in* Crassostrea hongkongensis* [129]. This unusual transcription pattern was attributed to prolonged exposure to heavy metals. This raises further question mark on the validity of HSPs as a universal biomarker of stress.

## 5. Closing Remarks

All studies, whether supporting or contradicting the validity of HSPs as biomarker of effect or exposure, are useful for establishing the biological exposure limits of toxicants and in creating awareness about their biological effects. Though some contradictory studies reject the application of HSPs as biomarkers in various fields and situations, these actually, from one perspective, also aid the more appropriate application of HSPs as biomarkers elsewhere by suggesting the types or organisms or conditions where HSPs may be less suitable or useful. Furthermore, these studies also suggest certain new fields of research in proposed model organisms for ERA. For example, the search for self- and cross-tolerance and seasonal, individual, sex, and gender based variations in the levels of HSP70/HSP60 alone as well as in combination with various environmental factors (temperature, salinity, pH, oxygen/hypoxia, etc.) with emphasis on toxicant uptake, accumulation, detoxification, synergistic effects, threshold levels, and induction kinetics of HSPs in proposed models are all areas of valid further examination. Further, “suit of biomarkers in a set of organisms” should also be investigated under these guide lines before suggestion of the application of such for environmental risk assessment. Furthermore, the relative sensitivity of northern or western blotting should also be examined to authenticate the either techniques for biomarkers studies in proposed model organisms. Though the levels of HSPs increase in dose- and time-dependent manner, this is only up to a particular limit of each toxicant after which their expression decreases. This aspect has a high potential for the confounding of results. Therefore, evaluating studies should be initially conducted for different time intervals along with the running of controls. In conclusion two main objections remain. The first is that the synergistic effects of toxicants with each other and also with environmental factors are strong enough to confound the validity of HSPs as a biomarker of toxicity, exposure, or effect. This is as the environment operates as a “whole,” as a dynamic and fluctuating system, and as such its factors fail to operate in isolation or in neat sequences. The second difficulty is in their expression in response to variety of stress conditions that are not related to toxicity and, therefore, in how to isolate and ascertain the cause from the response.

From recent studies, it becomes evident that HSPs show variable response in different organisms and even to different stressors. Hence, before application as biomarker, their response should be carefully checked against different stressors. Moreover, false interpretations could be drawn if solely HSPs are used as biomarkers. However, more integrated approach could be more conclusive.

Thus, it is clear that, at the present, studies of heat shock proteins remain so far unable to give more than an overall general picture of the environment instead of more exact information regarding a particular toxicant or pollutant. A much more systematic study is required, we suggest, with the focus upon broadening the testing of a range of potential toxicants and environmental factors for a limited number of key target species. Furthermore, there is need of search for target organs specific for a particular toxicant. With such a systematic and focused approach such biomarkers could yet possibly be elevated, when applied in sets, from the current general indications they provide to becoming techniques which yield much more specific, useful, and accurate data readily applicable for environmental management.

## Figures and Tables

**Table 1:** Biomarkers proposed for environmental risk assessment.

Name of biomarker	Reference
Esterases	[130]
Polytene chromosomes of chironomids (Diptera)	[131]
HSP60, HSP70, alpha B-crystallin homologue, lipid peroxide, total glutathione level, ubiquitin, mitochondrial manganese superoxide dismutase, metallothionein, and cytochrome P450 2E homologue	[93, 122, 123]
Numbers of macrophages in liver tissue, changes in various blood parameters	[105]
Histological and ultrastructural markers, lysosomal membrane stability of coelomocytes, histidine	[51, 132]
Catalase activity	[78]
Metabonomics analysis using NMR techniques	[133]
P-glycoprotein, major vault protein, topoisomerase-II	[103]
Acetylcholinesterase inhibition and imposex	[134]
Digestive enzymes, glycolytic enzymes, and cellular energy allocation	[124]
Apoptosis in marine sponges and spiders	[135, 136]

**Table 2:** HSP70 levels in clams during adaptation to various salinities∗.

Salinity (ppt)	HSP70 (relative density ×10^3^)		
	After 24 h	After 24 h	After 24 h
0.1	17.91	17.41	14.59
3	17.72	23.92	38.08
6	27.34	39.25	35.82
10 (ambient)	31.05	26.20	17.80
14	19.56	36.10	32.6
27	37.53	31.08	35.56

*Clams collected from Martinez marina (salinity: 10 ppt). Values represent relative density arbitrary units of bands detected by western blotting of pooled samples of five clams* Potamocorbula amurensis*, with no significant differences in temperature but salinity increasing in gradient manner in the sampling sites (Werner and Hinton, 2000 [106]).
