# Corrigendum to “Synergistic Effects of Toxic Elements on Heat Shock Proteins”

**DOI:** 10.1155/2015/204057

**Published:** 2015-04-23

**Authors:** Khalid Mahmood, Saima Jadoon, Qaisar Mahmood, Muhammad Irshad, Jamshaid Hussain

**Affiliations:** ^1^Department of Biology, Government Post-Graduate College Asghar Mall, Rawalpindi, Pakistan; ^2^Department of Natural Resource Engineering and Management, University of Kurdistan-Hawler Erbil, Kurdistan Region, Iraq; ^3^Department of Environmental Sciences, COMSATS Institute of Information Technology, Abbottabad 22060, Pakistan

In Figure 5 in the paper titled “Synergistic Effects of Toxic Elements on Heat Shock Proteins,” we cited wrong reference, [120] K. S. Ali, L. Dorgai, M. Ábrahám, and E. Hermesz, “Tissue- and stressor-specific differential expression of two hsc70 genes in carp,”* Biochemical and Biophysical Research Communications*, vol. 307, no. 3, pp. 503–509, 2003. The correct citation of Figure 5 is “A. Ali, P. H. Krone, D. S. Pearson and J. J. Heikkila “Evaluation of stress-inducible hsp90 gene expression as a potential molecular biomarker in xenopus laevis,”* Cell Stress and Chaperones*, vol. 1, no. 1, pp. 62–69, 1996.”

